# Exploring tannin structures to enhance enzymatic polymerization

**DOI:** 10.3389/fchem.2025.1555202

**Published:** 2025-03-04

**Authors:** Romina Romero, Tihare Gonzalez, Bruno F. Urbano, Cristina Segura, Alessandro Pellis, Myleidi Vera

**Affiliations:** ^1^ Departamento de Química Analítica e Inorgánica, Facultad de Ciencias Químicas, Universidad de Concepción, Concepción, Chile; ^2^ Departamento de Polímeros, Facultad de Ciencias Químicas, Universidad de Concepción, Concepción, Chile; ^3^ Unidad de Desarrollo Tecnológico, Universidad de Concepción, Coronel, Chile; ^4^ Dipartimento di Chimica e Chimica Industriale, Università degli Studi di Genova, Genova, Italy

**Keywords:** tannins, enzymatic polymerization, laccase, Py-GC/MS, pine bark, flame retardancy

## Abstract

The enzymatic polymerization of biomass-derived polyphenols presents a sustainable approach to producing advanced materials. However, the structural diversity and incomplete characterization of tannins pose challenges to optimizing the process. This study investigates how tannin composition and the presence of phenolic and non-phenolic compounds in aqueous *Pinus radiata* bark extracts influence laccase-catalyzed polymerization and the resulting material’s thermal and structural properties. The extracts were characterized using proximate and ultimate analysis, Py-GC/MS, FT-IR, TGA, and phenol content analysis before polymerization with *Myceliophthora thermophila* laccase (MtL). Structural and thermal analysis of the polymers revealed significant transformations driven by enzymatic oxidation. Tannin extracts rich in resorcinol and low in carbohydrates and less polar compounds produced highly cross-linked polymers with exceptional thermal stability, retaining 86% residual mass at 550°C. These findings demonstrate that tannin composition plays a key role in polymerization efficiency and material performance. The resulting thermally stable polymers offer potential applications in flame retardancy and sustainable material development, providing a promising pathway for biomass valorization.

## Graphical Abstract

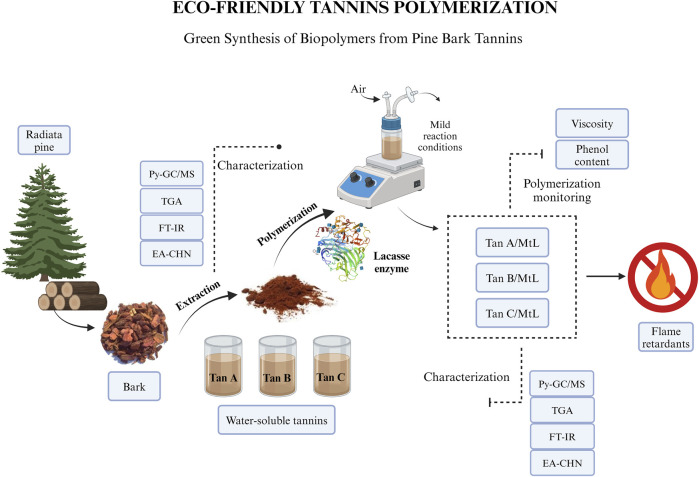

## 1 Introduction

Environmental concerns associated with the utilization of fossil-based plastics, along with new stricter regulations and promising opportunities presented by emerging technologies, have fueled interest in developing biopolymers as a sustainable alternative to drive the growth of the plastics industry. According to the “Global Market for Bioplastics and Biopolymers 2023” report, the bioplastics and biopolymers market is forecasted to reach USD 27.3 billion by 2027 (Company, 2024), highlighting a significant opportunity for the advancement of new biomaterials derived from the responsible utilization of natural resources.

**FIGURE 1:**
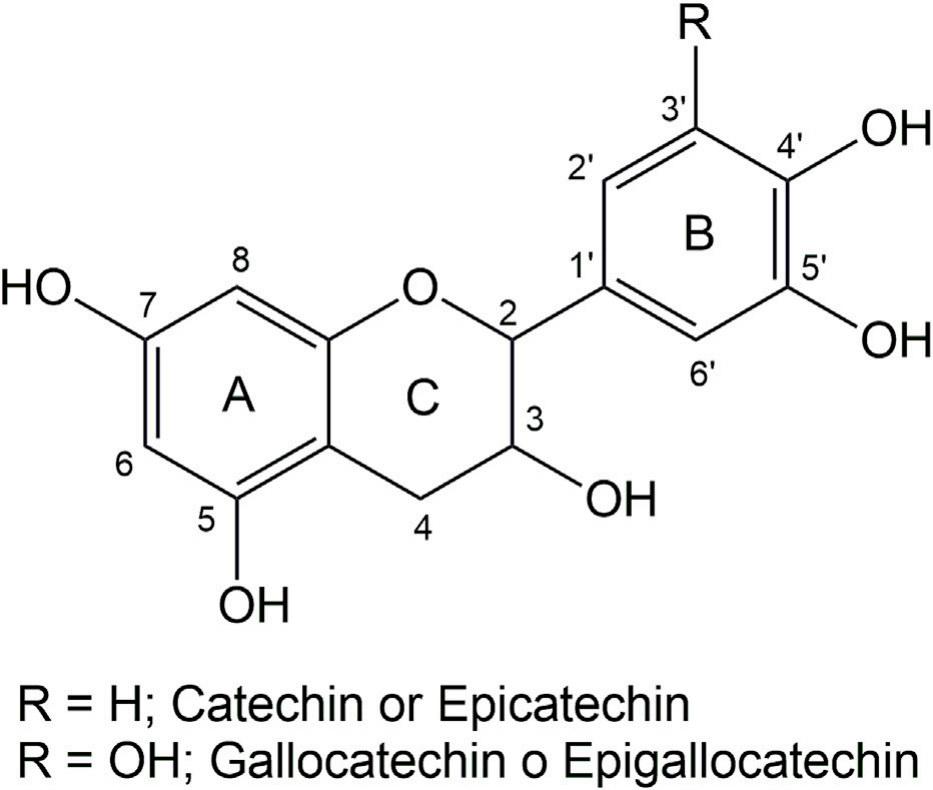
Chemical structure of monomers of condensed tannins.

Tannins are the second most abundant source of natural polyphenols in nature (after lignin). They are an excellent renewable raw material to produce bioplastics due to their abundance in nature, ubiquity, cost-effectiveness, and chemical versatility (Vera et al., 2023a). These macromolecules exhibit remarkable antioxidant, antibacterial, and antimicrobial properties (Cano et al., 2021). Tannins can be classified into four categories: condensed tannins (CT), hydrolyzable tannins (HT), phlorotannins (PhT), and complex tannins (Vera and Urbano, 2021). Condensed tannins represent approximately 90% of the global commercial tannin production, characterized by oligomeric structures comprising monomers like catechin, epicatechin, gallocatechins, or epigallocatechins (Figure 1) (Vera and Urbano, 2021).

The bark of *Pinus radiata* is an important source of tannins. Known as the most widely cultivated exotic softwood globally, *P. radiata* stands out for its exceptional productivity and versatility in various industries, including construction and pulp and paper. This species is predominantly found in Australia, Chile, New Zealand, South Africa, and Spain (Mead, 2013).

Pine bark, considered a by-product of the agroforestry industry, has high concentrations of condensed tannins (12-20 wt. %) (Garcia et al., 2016). Tannins have gained widespread use in industries like leather production, adhesives, and medicine (Ayalew and Wodag, 2023; Liman et al., 2022). Another avenue for valorizing tannins lies in harnessing their remarkable polyphenolic characteristics to create tannin-based materials tailored for specific applications. Recent breakthroughs have showcased the utilization of tannins in synthesizing diverse polymer types through reactions with isocyanates, epoxy resins, amines, formaldehyde, and many other molecules (Vera and Urbano, 2021; Chen et al., 2022; Zhang et al., 2022). In these synthetic procedures, tannins play key roles as functional additives and cross-linking agents, as well as in grafting polymer synthesis (Vera et al., 2023b; Zhang et al., 2023). Incorporating tannins into polymeric structures offers a range of benefits, including enhanced UV radiation resistance, increased crosslinking, improved mechanical properties, greater water resistance, cost-effectiveness, biodegradability, and antioxidant, antifungal, and antimicrobial properties (Črešnar et al., 2022).

The structural and molecular weight (Mw) diversity of tannins leads to highly polydisperse materials from extracts, resulting in often non-reproducible processes. As a result, many studies resort to utilizing well-defined and controlled commercial monomers derived from tannin extraction and purification processes, such as tannic acid, catechol, resorcinol, and catechin (Vera and Urbano, 2021; Aktaş et al., 2003; Kurisawa et al., 2003; Borah and Karak, 2022). However, using these monomers can be expensive when scaling up the technology, posing challenges in replacing them as alternatives to fossil-based polymers (de Hoyos-Martínez et al., 2019). A more economically feasible approach involves leveraging unpurified tannin extracts and conducting a thorough characterization to ensure property’s consistency of the resultant materials.

Various chemical, physical, and enzymatic approaches have been explored to synthesize tannin-based polymeric materials (Vera and Urbano, 2021). In recent years, enzymatic polymerization has garnered significant attention with rapid technological advancements. Compared with chemical methods, enzymatic polymerization possesses higher selectivity, occurs under mild conditions, without toxic organic solvents, and with processes requiring less energy input (Agustin et al., 2021). Laccase enzymes (diphenoloxidases, EC 1.10.3.2) are oxidases that allow valorizing tannins by polymerization or grafting reactions. These enzymes are widely known for their ability to catalyze the oxidation of various aromatic compounds (especially phenols) using molecular oxygen as an electron acceptor and generating water as the only by-product (Vera et al., 2024).

The electron abstraction capability of laccases, coupled with the concurrent oxidation of phenolic compounds to generate reactive species, serves as the foundation for polymerization reactions (Nyanhongo et al., 2010). In nature, laccase enzymes play crucial roles in lignin’s polymerization and depolymerization processes. Consequently, extensive research has been conducted on the polymerization of lignin derivatives using laccases (Vera et al., 2023a; Johansson et al., 2012). Similarly, oxidation reactions involving phenolic compounds derived from tannins have shown promising properties in developing functional polymers (Vera et al., 2024). Despite these advancements, further enhancements in this research domain are essential. The proposed mechanism for enzymatic polymerization mediated by laccase involves the formation of phenoxy radicals through hydrogen extraction, followed by radical polymerization reactions (Rättö et al., 2004).

While numerous studies have highlighted the potential of incorporating tannins into polymeric matrices through laccase-mediated processes, there is an urge to elucidate the extracts used and the corresponding chemical transformations, employing a thorough characterization of the process (Vera and Urbano, 2021).

The main objective of this research is to explore the relationship between the structural properties of distinct pine bark tannins and the efficacy of laccase-assisted tannin oxidation/polymerization. Additionally, this study aims to gain insights into the chemical alterations of tannins post-treatment with the enzyme *Myceliophthora thermophila* laccase (MtL) via an eco-friendly and sustainable approach. To achieve this objective, various aqueous extracts of *P. radiata* bark sourced from the Bio-Bío region of Chile were subjected to comprehensive characterization using proximate and ultimate analysis, Pyrolysis-Gas Chromatography/Mass Spectrometry (Py-GC/MS), Fourier Transform Infrared Spectroscopy (FT-IR), Thermogravimetric Analysis (TGA), and phenol content analysis. These extracts, primarily composed of water-soluble tannins, underwent enzymatic oxidation/polymerization facilitated by the MtL enzyme. The resulting polymers/oligomers underwent further characterization to unveil the structural modifications induced by the laccase reaction and explore the potential applications of these materials.

## 2 Materials and methods

Laccase (EC 1.10.3.2) from *M. thermophila* was provided by Novozymes, Denmark. Industrial tannins were acquired from Bioforest and Unidad de Desarrollo Tecnológico (UDT). Tannic acid, 2,2′-azino-bis(3-ethylbenzothiazoline-6-sulfonic acid) (ABTS), Folin-Ciocalteu (FC) reagent, 2-methoxyethanol (99.8%), sodium carbonate (Na_2_CO_3_, ≥99.5%, Merck), bovine serum albumin (BSA), Bradford reagent, the reagents for buffers solutions (citric acid and disodium hydrogen phosphate) and all other reagents were purchased in analytical grade by Merck (Chile) and used as received.

### 2.1 Tannins characterization

The tannins used were extracted from *P. radiata* bark obtained from 15 to 20-year-old forest plantations in the Bio-Bío region (Chile). Three types of aqueous tannin extracts, Tan A, B, and C, were used. All types of tannins were obtained on a pilot plant scale (Technological Development Unit, UDT-UdeC, Coronel, Chile). In this study, the tannins used correspond to the soluble fraction. This fraction was obtained following the methodologies detailed below, achieving yields ranging between 3%–5%, which is consistent with values reported in the literature (Bocalandro et al., 2012).

For Tan A extractions, the solid/liquid ratio was set at 1:10 (w/w) and aqueous solutions of sodium sulfite or sodium hydroxide were used, with an extraction temperature of 90°C. The materials were combined with water at room temperature, heated, and sodium sulfite and sodium hydroxide solutions were added after the temperature reached 90°C. After 1 h of extraction, the suspension was vacuum filtered, the solid residue was washed under water flow (García et al., 2016; Case et al., 2014). After this procedure, a final extraction with methanol to remove higher molecular weight carbohydrates and less polar compounds was developed. Finally, the aqueous extract solutions were dried in a spray dryer.

On the other hand, Tan B was obtained from pine bark extraction with a 75% methanol/water mixture at a 1:6 mass/volume ratio (Case et al., 2014). The extraction was carried out at 120°C for 2 h. The water-soluble solid fraction was collected and dried in a spray dryer. Afterward, the volatile solvent was removed under reduced pressure (5.0 kPa) at room temperature. Tan C was extracted similarly to Tan A, without the last step of a final extraction with methanol (García et al., 2016; Case et al., 2014).

Initially, the materials were ground and sieved to achieve a particle size of 0.6 to 1.0 mm, followed by oven-drying at 40°C. Proximate and ultimate analyses were performed to determine the composition of all the studied tannins.

#### 2.1.1 Proximate and ultimate analysis of tannins

The materials were ground and sieved to obtain a particle size between 0.6–1.0 mm and oven-dried at 40°C. Proximate and ultimate analyses were performed to evaluate the composition of the various tannins, which were compared between them. According to Pinto et al., moisture, ash, volatile matter, and fixed carbon content were determined (Pinto et al., 2018). Elemental analysis (EA) of C, H, and N was carried out using a Leco (628 CHN3607, United States) instrument, with the oxygen content calculated based on the difference in elemental composition.

#### 2.1.2 Fourier transformed infrared spectroscopy analysis (FTIR)

FT-IR spectra of tannins (Tan) and tannin polymers (Tan/MtL) were acquired using a Fourier-Transform Infrared Spectrometer (FTIR, NICOLET model Nexus equipped with a DTGS-KBr detector). Spectra were taken in the range of 4,000–400 cm^−1^. FT-IR measurements were conducted on a consistent sample size of 1.00 ± 0.002 mg to ensure data comparability. In addition, the data was post-processed, applying a normalization to 1,976 cm^−1^ because it was a signal that had no changes in all the raw analyzed spectra.

#### 2.1.3 Thermogravimetric Analysis (TGA)

The thermal properties of tannins (Tan) and laccase-polymerized tannins (Tan/MtL) were studied by thermogravimetric analyses, TGA, using a NETZCH TG 209F1 Iris 220-12-0045-L equipment. Samples weighing between 3 and 5 mg were subjected to analysis following a temperature program comprising: i) step 1 isothermal at 50°C for 30 min, ii) step 2 isothermal at 80°C for 15 min, iii) step 3 temperature ramp at 10°C·min^−1^ from 100°C to 550°C. All procedures were conducted under a nitrogen atmosphere with a continuous gas flow of 20 mL min^−1^.

#### 2.1.4 Analytical micropyrolysis (Py-GC/MS)

The volatiles extracted from different tannins isolated from *Pinus radiata* bark (Tan) and tannin polymers (Tan/MtL) were examined at 550°C to evaluate their primary decomposition products at this specific temperature. Simultaneously, an exploratory investigation of the chemical composition of volatiles from the various tannins at 550°C was conducted to identify the critical differences in the primary thermal decomposition products of each phenolic source under uniform experimental conditions. The analytical pyrolysis experiments were performed using an isothermal micropyrolysis unit (EGA-PY 3030D, Frontier Laboratories) coupled with a gas chromatograph (GC, 2010, Shimadzu) equipped with a mass spectrometry detector (QP 2010, Shimadzu).

Approximately 100 μg of the sample was weighed using a microbalance (Excellence Plus XP6 model, Mettler Toledo) and introduced into the preheated furnace via a manual shot sampler in the micropyrolysis unit. A 20°C·s-1 heating rate was applied until the pyrolysis temperature was reached under a helium flow of 1.00 mL min^−1^. The volatile compounds were separated and analyzed using a GC/MS system with an HP-5MS column (length 30 m; internal diameter 0.25 mm; film thickness 0.25 μm, Agilent Technologies, United States).

The injector and detector temperatures were maintained at 250°C and 280°C, respectively. The initial GC oven temperature was set at 45°C and held for 4 min before ramping to 280°C at 3°C·min^−1^, which was sustained for 40 min. A split ratio of 15:1 was employed for injection. While it is recognized that the Py-GC/MS technique does not provide a quantitative analysis of detected compounds, it offers a semi-quantitative analysis based on the relative content of each compound. The mean values and corresponding standard deviations of peak areas for each identified compound were calculated based on the peak area (%) of compounds detected by the mass selective detector in the pyrolysate, enabling interpretation (Arteaga-Pérez et al., 2018; Cabrera-Barjas et al., 2023). Peak identification was facilitated by utilizing the MassFinder 4 software and the NIST (2014) mass spectral library. The identified compounds were then classified into ten categories: light volatiles, acids, ketones/aldehydes, phenols, carbohydrate-derived compounds, aromatics, hydrocarbons, fatty acids, and alcohols.

It should be noted that while Py-GC/MS cannot determine the absolute content of compounds formed during pyrolysis, a linear relationship between the chromatographic peak areas corresponding to each compound and their concentration can be established as a semi-quantitative approach. The 30 most intense peaks in all chromatograms were selected, and the area % count was normalized to facilitate a valid comparison between the tannin samples (Equation 1) (Arteaga-Pérez et al., 2018). Additionally, the relative amount of CO_2_ was eliminated from the analysis.

$$S_{i}=100\times \left(\frac{{Peak\;Area}_{i}}{\sum_{i=1}^{n}{Peak\;Area}_{i}}\right)$$ (1)

### 2.2 Laccase characterization

#### 2.2.1 MtL purification

The MtL enzyme underwent a thorough purification process to eliminate impurities and external agents. This involved washing the enzyme five times through ultrafiltration using Viva Spin membranes with a molecular weight cutoff of 30 kDa HY, following a protocol previously documented by Vera et al. (2019). All centrifugation steps were meticulously conducted at 4°C, and activity assessments were carried out before and after purification. The purity and molecular weight of the enzyme were then assessed using sodium dodecyl sulfate-polyacrylamide gel electrophoresis (SDS-PAGE).

#### 2.2.2 Determination of laccase activity

The activity of the MtL laccase enzyme was evaluated based on a method outlined by Vera et al. (2020), with slight modifications. Briefly, the oxidation of ABTS to its cation radical was monitored at 420 nm for 5 min at 25°C using an Agilent Epoch 2 spectrophotometer (Agilent Technologies, United States). These measurements were performed at pH 7 in a buffer solution of 0.1 M citrate/0.2 M phosphate. The activity was expressed in U, defined as the enzyme required to oxidize 1 µmol of substrate per minute. All measurements were performed in triplicate and reported with their respective standard deviations.

#### 2.2.3 Protein concentration

The protein content used in the polymerizations was determined through the Bradford assay method, following the established protocol by Huber et al. (2016a). Briefly, 200 µL of a diluted BioRad solution 1:5 MQ water was mixed with 10 µL of a diluted sample and incubated for 5 min at 400 rpm. Subsequently, the absorbance was measured at 595 nm using an Agilent Epoch 2 spectrophotometer (Agilent Technologies, United States). The protein concentration was determined using a bovine serum albumin standard curve in the 0.0125–0.5 mg mL^−1^ range. All measurements were made in triplicate.

### 2.3 Polymerization of tannins using laccase

A dedicated study investigated the polymerization of tannins with the MtL enzyme. Initially, tannins were dissolved in a citrate/phosphate buffer at pH 7 to achieve a final 10% total dry substance (TDS) concentration (Vera et al., 2024). The enzymatic reaction was initiated by introducing 500 U·mL^−1^ of MtL. Polymerization was carried out at 50°C with continuous stirring at 250 rpm, accompanied by aeration to maintain oxygen levels in the medium. Regular sampling every 15 min allowed for monitoring phenol content, viscosity, and fluorescence intensity throughout the reaction.

#### 2.3.1 Viscosity measurements

Viscosity measurements of the reaction samples were conducted using a parallel plate rheometer (DHR-3, TA Instruments) with a Peltier plate set at 20°C. The frequency was set to 1 Hz with a strain of 10%. The plate diameter was 20 mm, and the gap between the parallel plates was maintained at 0.15 mm. Each sample was analyzed in triplicate to ensure accuracy and consistency.

#### 2.3.2 Phenol content measurements

The total concentration of phenolic groups was determined using the Folin-Ciocalteu (FC) method following the procedure described by Bartolome et al. (2022). To measure phenol content, 20 µL of the filtered and diluted sample was mixed with 60 µL of FC reagent and incubated at room temperature for 5 min. Subsequently, 200 µL of MQ water and 120 µL of a sodium carbonate solution (20% w/v) were added. The reaction mixture was stirred at 800 rpm for 2 h, followed by absorbance measurement at 760 nm using a plate reader Agilent Epoch 2 spectrophotometer (Agilent Technologies, United States). A calibration curve was established using catechol as a standard reference. All measurements were performed in triplicate for statistical reliability.

#### 2.3.3 Water solubility test

The solubility of the samples was evaluated in water after the enzymatic reaction. For this purpose, the samples were dried at 35°C for 24 h. Then, approximately 10 mg of sample was immersed in 3 mL of distilled water and shaken at 21°C for 24 h. Subsequently, the samples were removed from the reaction medium and dried in an oven to constant weight at 35°C to determine the solubilized mass. The solubility in water was determined by the Equation 2 (Vera et al., 2024).

$$Water\;solubility\;\left(\%\right)=\left(\frac{W_{i}-W_{f}}{W_{i}}\right)\times 100$$ (2)

where *W*_*i*_ denotes the initial weight of the sample before immersion in distilled water, and *W*_*f*_ refers to the dry weight of the sample after immersion.

## 3 Results and discussion

### 3.1 Feedstock characterization

Aqueous fraction of 3 types of tannins extracted from the bark of 15–20 years old *P. radiata* trees planted in controlled crops (Bio-Bío region, Chile) were studied. The three types of tannins, Tan A, Tan B, and Tan C underwent characterization before, during, and after enzymatic polymerization to evaluate the impact of their chemical structures on polymerization efficiency.

The three types of tannins showed a low inorganic content of <0.20 wt. %. Tan B displayed the highest volatile matter content at 73.4 wt. % and fixed carbon at 18.4 wt. % (Table 1), while Tan C showed the lowest volatile matter content and the highest fixed carbon content, suggesting a more complex matrix possibly constituted by components of higher molar mass (M_W_, molecular weight) and with a consequent higher biochar generation in this biomass (Klasson, 2017).

**TABLE 1:** Proximate and ultimate analysis of tannins.

Proximate analysis (db, wt%)	Tan A	Tan B	Tan C
Moisture	12.00 ± 0.10	8.04 ± 0.08	6.99 ± 0.11
Ash	0.11 ± 0.02	0.18 ± 0.02	0.19 ± 0.01
Volatile matter	68.40 ± 0.61	73.40 ± 0.22	61.50 ± 0.4
Fixed carbon^a^	19.49 ± 0.95	18.40 ± 0.44	31.30 ± 0.75
Ultimate analysis (daf, wt%)			
C	46.3 ± 0.1	44.9 ± 0.1	52.8 ± 0.3
H	4.64 ± 0.06	4.27 ± 0.19	5.41 ± 0.01
N	0.408 ± 0.019	0.326 ± 0.004	0.190 ± 0.033
O^a^	48.7 ± 0.2	50.5 ± 0.6	41.6 ± 0.8

^a^ By difference; db, dry basis; daf, dry and ash-free.

In terms of ash content, Table 1 shows a lower ash content for Tan A (0.11 wt%) and higher ash content for Tan B (0.18 wt%) and Tan C (0.19 wt%). Previous studies on enzymatic polymerization have associated higher ash content with a reduced tendency for enzymatic polymerization. Thus, based on ash content alone, Tan A would be expected to have a higher propensity for polymerization through laccase enzyme action (Mayr et al., 2021).

The results obtained from EA-CHN were used to calculate the atomic H/C and O/C ratios, indicating the samples’ bonding arrangement and polarity. According to the results obtained (Table 1), it could be observed that all the tannins tested have similar H/C ratios, with values of approximately 0.10 for all cases (0.100, 0.102, 0.095; for Tan A, Tan B, and Tan C, respectively). These values were based on previous reports in the literature on bark tannins (Case et al., 2014). However, the O/C ratios drastically differ, showing values of 1.05, 1.12, and 0.79 for Tan A, Tan B, and Tan C, respectively. The O/C ratio measures the abundance of oxygen-containing functional groups and can be used to estimate the hydrophilicity of biomass (Cui et al., 2016). These results suggested that Tan C may contain fewer oxygenated functional groups than Tan A and Tan B, potentially indicating a higher presence of hydrocarbon structures. This is consistent with the proximate analysis, which showed a lower volatile content for Tan C, suggesting a possible higher component condensation. Conversely, the higher O/C ratio of Tan B suggests a composition rich in oxygenated and lower molecular weight compounds, which is consistent with its higher water solubility compared to Tan A and Tan C. This difference can also be observed experimentally in the water solubility of these tannins, where a higher solubility of Tan B > Tan A > Tan C could be observed, which could be related with the O/C ratio, where a higher hydrophilicity would be expected for Tan B.

**FIGURE 2:**
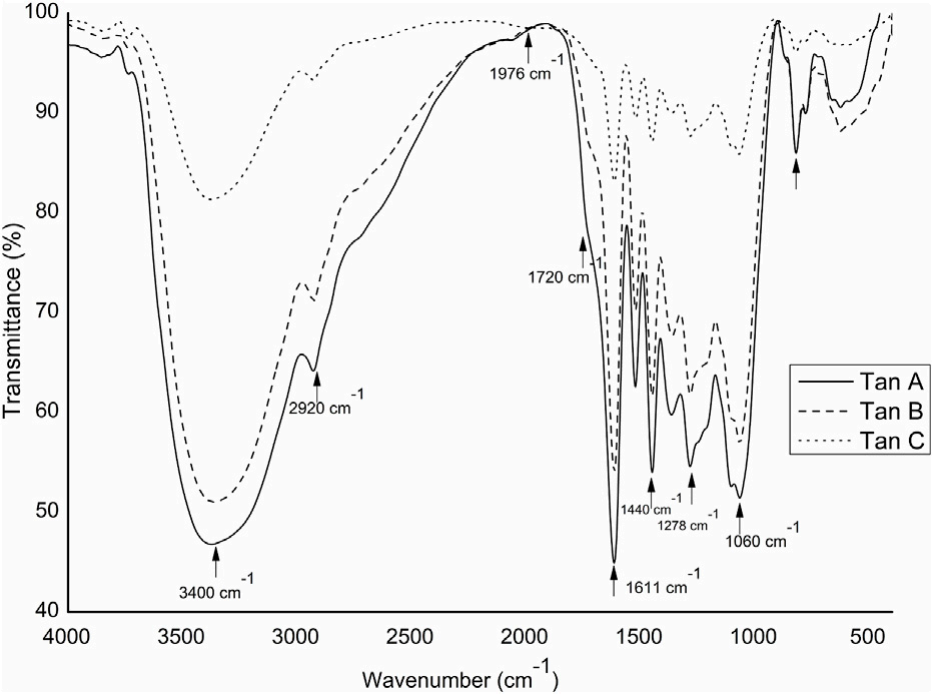
FT-IR spectra for the tannins employed: Tan A, Tan B, and Tan C

Mid-infrared spectra of the three tannins in their raw form were analyzed. The spectra underwent baseline correction and normalization at 1976 cm^−1^ to facilitate comparability and minimize errors in KBr tablet preparation. The FT-IR spectra are presented in Figure 2.

A distinct and broadband around 3,400 cm^−1^ was observed in all three tannins, attributed to the stretching vibration of the -OH group in both aliphatic and phenolic structures. Variances in absorption intensities within this region indicated a decreasing presence of these groups, with Tan B exhibiting higher intensity than Tan A and Tan C. Minor peaks at 2,920 and 2,840 cm^−1^ originated from the–CH stretching vibration in aromatic methoxy groups, as well as methyl and methylene groups. Small peaks at 2,920 and 2,840 cm^−1^ originate from –CH stretch vibration in aromatic methoxy groups and methyl and methylene groups of side chains (de Hoyos-Martínez et al., 2019). Signals at 1278 cm^−1^ corresponded to C-O bond tension and carboxylic group bonds, while pronounced peaks at around 1050–1060 cm^−1^ resulted from vibrational stretching of C–O bonds in phenols (Soto Fallas, 2001). Furthermore, signals at 1603 cm^−1^ and 1513 cm^−1^ were assigned to the deformation vibrations of the carbon-carbon bonds in the 1605–1496 cm^−1^ region in the aromatic structures of these types of tannins. On the other hand, the bands at 1720 cm^−1^ could be assigned to the -C=O stretching of aliphatic esters. Esters exhibit absorptions for symmetric and antisymmetric stretching of C-O-C in ranges from 1,050 to 1,300 cm^−1^ (Nikonenko et al., 2005).

**FIGURE 3:**
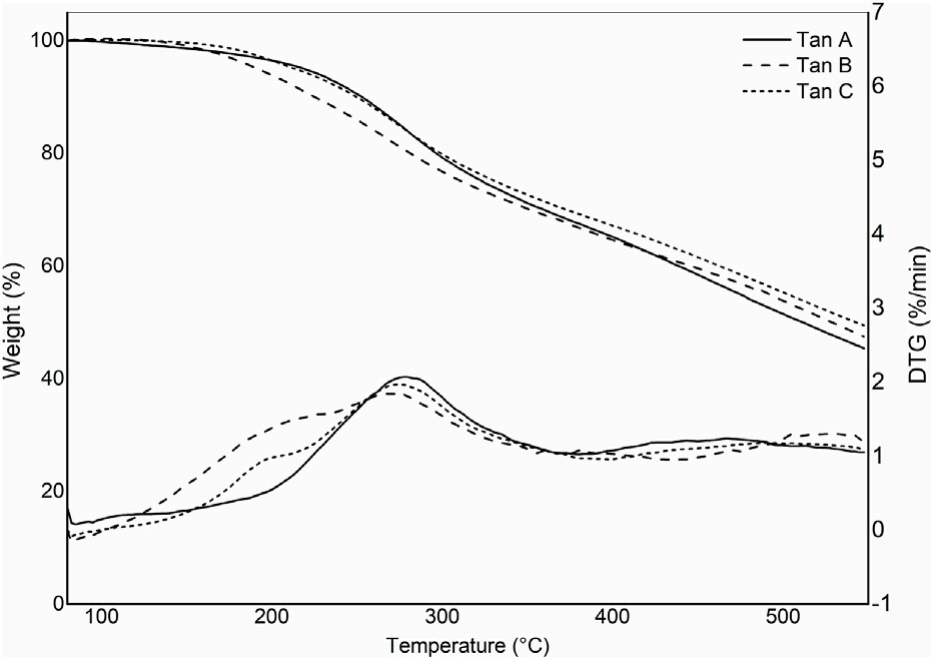
Thermogravimetric and DTG profiles for Tan A, Tan B, and Tan C.

Figure 3 shows the thermograms depicting the thermal decomposition of the investigated tannins. The thermal decomposition profiles show no significant variations in mass loss with increasing temperature, indicating similar compositions. A preheating program described in the methodology was implemented to avoid mass losses associated with water evaporation. As a result, the TGA profiles do not display mass losses below 100°C due to the evaporation of adsorbed water from the sample (Figure 3). To elucidate the stages of thermal degradation, Differential Thermal Gravimetric Analysis (DTGA) was performed, revealing distinct degradation events: the first occurring at approximately 200°C, characteristic of catechin present in the tannins (Gaugler and Grigsby, 2009), with a minimal weight loss of around 5%, as observed in Tan B and Tan C. A more significant weight loss is observed at approximately 280°C (ca 20%), corresponding to catechin degradation due to the breakdown of the C2-C1′ bond of the C and B rings (Figure 1.) (Pinto et al., 2018). The third weight loss occurs at around 460 °C for Tan A and Tan C and at 520°C for Tan B, attributed to the degradation of lignin, which has high thermal stability (Barros et al., 2023). At the end of the heating period, a substantial residual mass of around 50% is observed at 550°C, likely due to the vigorous char formation resulting from crosslinking reactions within the aromatic structure during the heating process (Sebestyén et al., 2019). These thermal degradation profiles align with previous studies on the thermal degradation of hydrolyzable tannins (Sebestyén et al., 2019). Based on these data and previous studies of the pyrolysis of tannins isolated from pine bark, where it was established that the highest amount of tannin-volatiles was found at 550°C, this temperature was chosen to perform the pyrolysis of tannins (Case et al., 2014).

**FIGURE 4:**
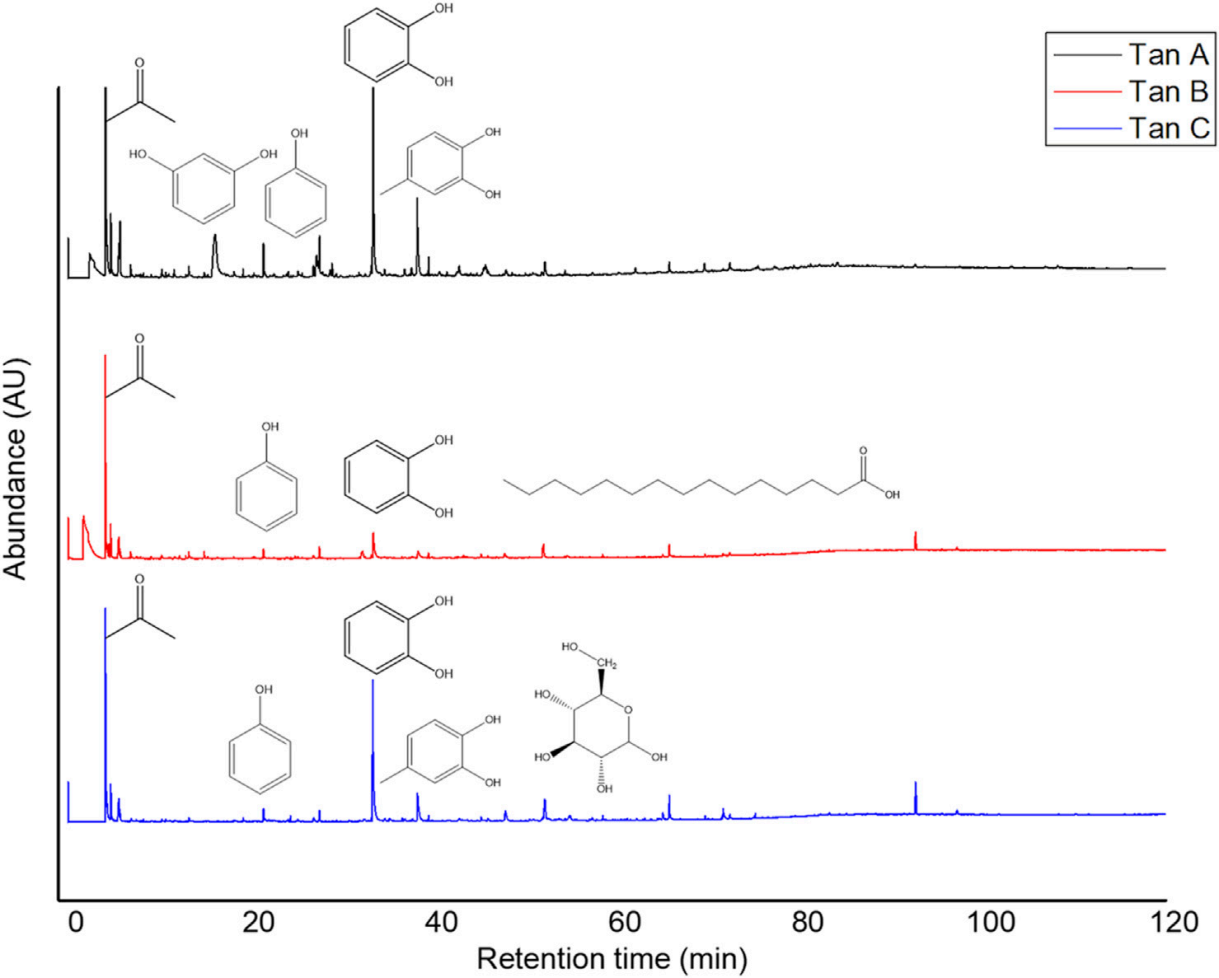
Py-GC/MS profiles for Tan A, B, and C.

The micropyrolysis assays were performed under identical experimental conditions for all tannins to determine the primary degradation products (550°C, 6 s of residence time). The Py-GC/MS chromatograms are presented in Figure 4. The relative standard deviation of the peak areas of the Py-GC/MS total ion chromatograms ranged from 5 to 15% when replicate experiments were conducted (in triplicate). The main detected products released from the fast pyrolysis of samples exhibited different profiles depending on the type of tannin.

**FIGURE 5:**
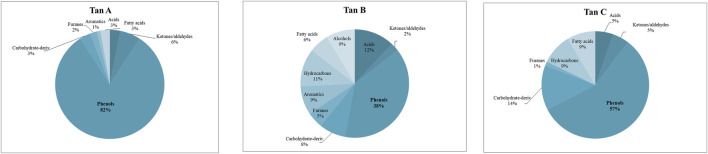
Primary decomposition product distribution for each tannin studied.

The identified compounds were then grouped according to their predominant functional group, and the relative content of each group was determined as a percentage of the total identified peak area (%). The identified compounds were categorized into ten groups: light volatiles, acids, ketones/aldehydes, phenols, carbohydrate-derived, aromatics, hydrocarbons, fatty acids, and alcohols. The chemical distribution is shown in Figure 5. According to the normalized data, the peak areas were transformed into area percentages, a type of semi-quantification in Py-GC/MS. Figure 5 highlights apparent differences in the relative contents for each of the analyzed tannin. Notably, Tan A exhibits a higher relative amount of phenolic components as primary products of thermal decomposition (82%).

On the other hand, Tan B has the lowest content of phenolic compounds (38%). However, this tannin shows a higher distribution of low molecular weight oxygenated compounds, consistent with the observations discussed in the proximate and ultimate analysis section.

It is important to note that, controversially, the FT-IR analyses show a higher content of -OH vibrations for Tan B than for Tan C. However, this aligns with the fact that this region is associated with aromatic and linear alcohols. In this context, Tan B is the only sample that shows linear alcohols structures among its primary thermal decomposition products. These may originate from the thermal decomposition of higher molecular weight linear alcohols or from cyclic structures that have broken down and possibly recondensed, which would explain the higher absorbance of this band in the infrared analysis (Figure 2).

Other differences can be observed in the content of compounds of lower polarity, such as hydrocarbons and fatty acids, where a higher content is determined in Tan C compared to Tan A and Tan B. Notably, Tan A does not contain any long-chain hydrocarbon compounds, whereas Tan B and Tan C contain 11% and 9%, respectively. The fatty acid content was 3%, 6%, and 9% for Tan A, Tan B, and Tan C, respectively.

### 3.2 Laccase characterization

The activity of the MtL enzyme was evaluated before and after purification to verify that the integrity of the enzyme was maintained during the ultracentrifugation steps. Our research group has previously assessed the activity of MtL enzyme at different pH and temperature values, and the maximum relative activity was reached at pH 7.0 (Vera et al., 2020). Therefore, pH 7.0 was chosen to carry out the polymerization reactions. Previous studies have shown that the catalytic activity when using ABTS is mainly related to the activity in the polymerization of phenols, suggesting that at this pH, the polymerization reactions of tannins would be more efficient than at other pH values (Vera et al., 2023a). In the activity study at different temperatures, the enzyme activity increased with increasing temperature. In this case, 50°C was chosen as the reaction temperature because, based on previous results (Vera et al., 2020), it is expected that the reaction will proceed faster than at room temperature while maintaining the integrity of the enzyme. Under the selected reaction conditions (pH 7°C and 50°C), the specific activity of the enzyme was 236.1 ± 1.9 U·mg^−1^.

### 3.3 Polymerization of tannins

All polymerization reactions of the different tannins were carried out with air bubbles, since it has been shown that in the polymerization of phenols catalyzed by laccases, oxygen increases the molecular weight of the polymers obtained enzymatically (Huber et al., 2016a).

**FIGURE 6:**
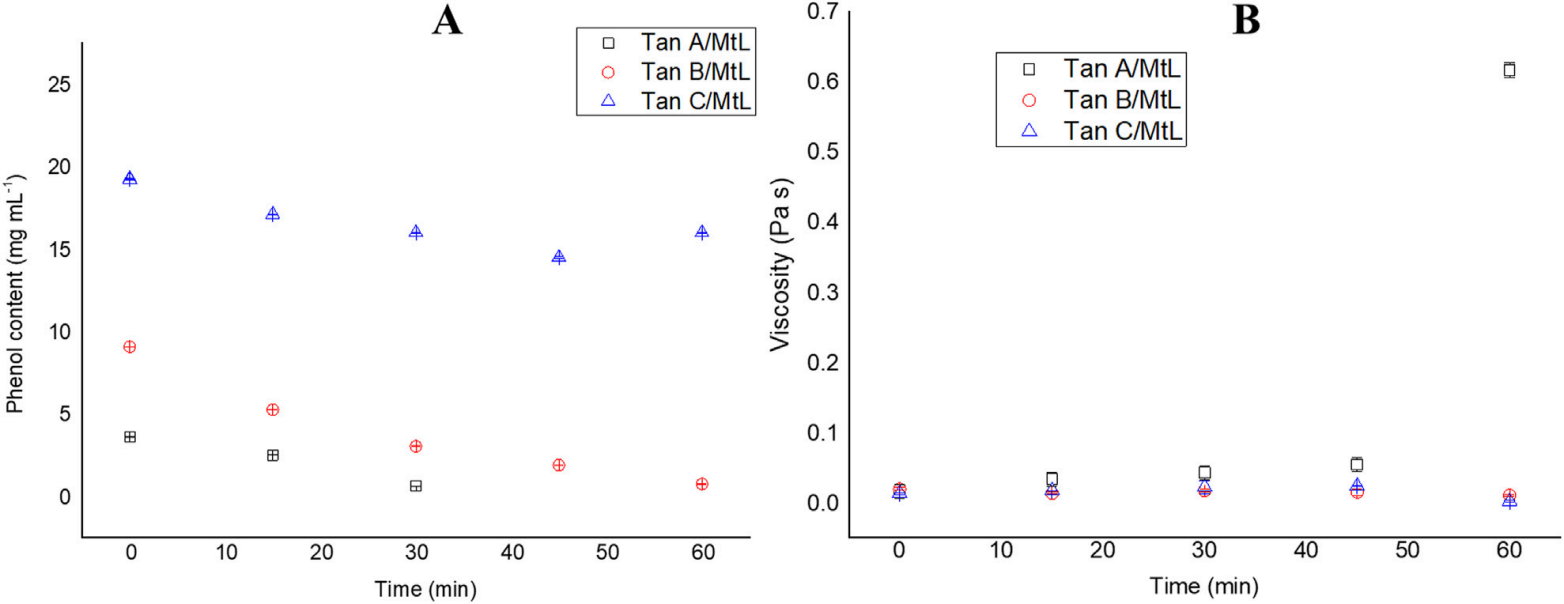
Changes in phenolic content **(A)** and viscosity **(B)** during the reaction of MtL with tannins.

The MtL enzyme reacted with the three types of aqueous tannin extracts while phenolic content and viscosity changes were monitored. Phenolic content is a quantitative technique that allows the degree of tannin modification to be tracked. This technique was used to monitor the polymerization of the different tannins. During the enzymatic reaction of tannins with laccase, a marked decrease in phenol content was observed in Tan A and Tan B throughout the polymerization. In contrast, only a slight decline was observed in sample C (Figure 6A). At the end of the enzymatic reaction, phenol content decreased by ∼100%, 92%, and 17% for Tan A, Tan B, and Tan C, respectively. These results indicate that the composition of each tannin and its polymerization efficiency are affected. The decrease in phenol content has been previously reported in polymerizations with lignosulfonates and has also been associated with an increase in the molar mass of the polymer being formed (Huber et al., 2016b). The decrease in phenol content can be attributed to the oxidative reactions catalyzed by laccases, in which phenols are oxidized, generating reactive radicals that can subsequently react to form semiquinones, quinones, or initiate self-coupling reactions to form new C-C, aryl-aryl, aryl-alkyl, or ether bonds that can undergo oligo- and polymerization (Kudanga et al., 2010). Thus, forming new bonds between the phenol radicals leads to a lower abundance of free phenol groups.

Figure 6B demonstrates that only Tan A exhibited a significant increase in viscosity following the enzymatic reaction, reaching 0.6 Pa s. This rise in viscosity is closely related to an increase in the molecular weight of oligomers and polymers formed, which is consistent with the high phenolic content of this tannin (Zerva et al., 2016). The significant increase in viscosity after 45 min of reaction could be attributed to the fact that in this reaction, which is carried out via free radicals, the oligomers begin to bind to give rise to the formation of polymers after 45 min of reaction. For this reaction, visually for Tan A, the formation of several nucleation points was observed where the resin began to form and in the last minutes of the reaction, these coalesced to give rise to the formation of a highly crosslinked resin. Similar trends have been observed for these enzymatic polymerizations with biomass (Vera et al., 2023a; Huber et al., 2016b). The polymers produced in this reaction, facilitated by laccase-mediated coupling of phenols, are known for their intense color, attributed to their expansive π system (Zerva et al., 2016). Conversely, Tan B showed no increase in viscosity, potentially due to the presence of low molecular weight phenols (as detected in the Py-GC/MS analysis) that form dimers or trimers that do not sufficiently increase viscosity upon reacting with the enzyme. Previous studies have reported this phenomenon on the laccase polymerization of phenols (Mayr et al., 2021). In contrast, Tan C, with its low phenol content, did not exhibit any detectable changes in viscosity, suggesting a lower reactivity towards enzymatic polymerization.

These results indicate a strong correlation between the high content of phenolic groups and the increase in viscosity and, consequently, molecular weight during laccase-mediated polymerization of tannins. Thus, high phenol content is a key characteristic for generating polymers with higher molecular weight and viscosity.

**FIGURE 7:**
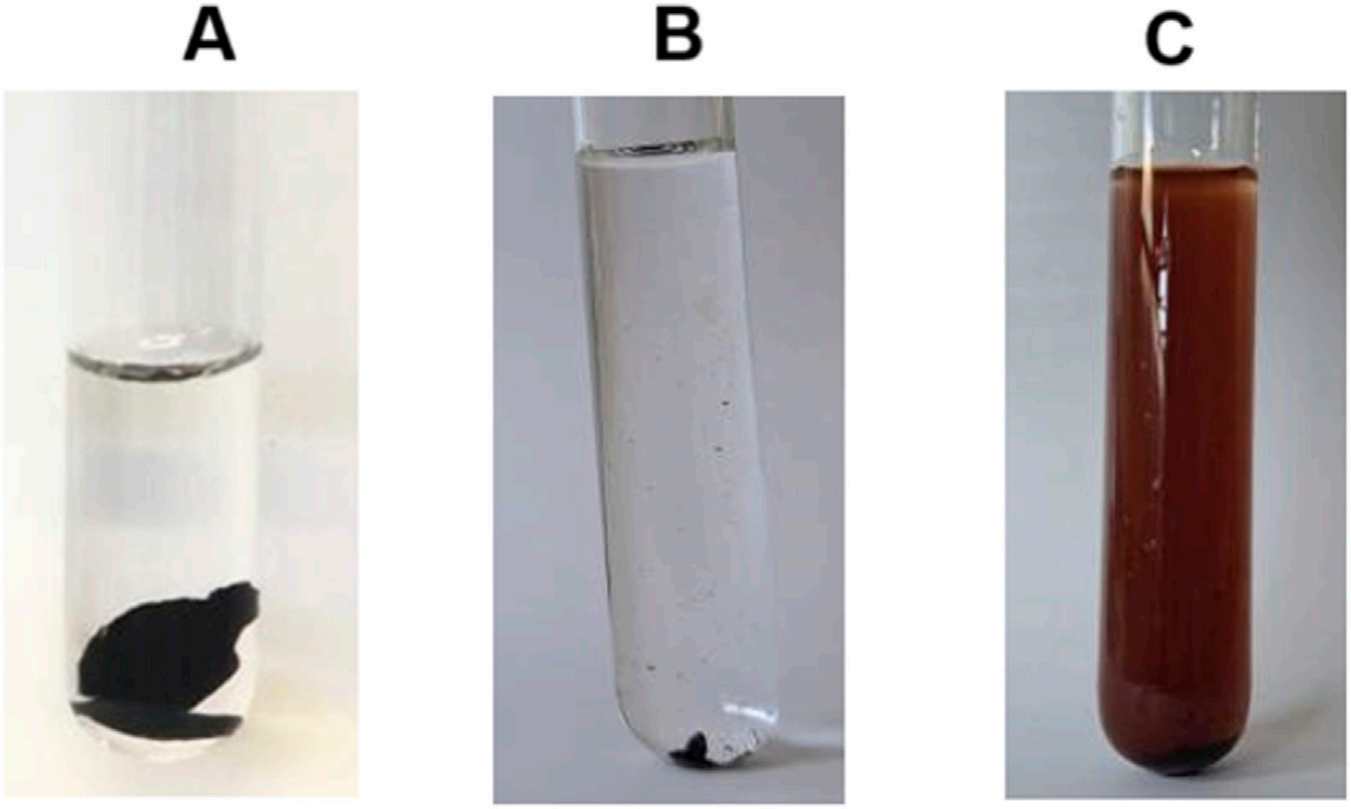
Water solubility test of Tan A/MtL **(A)**, Tan B/MtL **(B)**, and Tan C/MtL **(C)**.

Before the enzymatic reaction, all tannins were soluble in water. However, after the enzymatic reaction, Tan A was completely polymerized, becoming a highly cross-linked resin completely insoluble in water, so it was not possible to determine the molecular weight of the polymer obtained (Figure 7A). In the test of water solubility, for Tan B, precipitation of the reaction product was observed, which was also insoluble in water (Figure 7B). Finally, for Tan C, the reaction product presented solubility in water, which is very congruent with the results observed in the enzymatic reaction suggesting that for this tannin there was no polymerization and only oxidation (Figure 7C).

### 3.4 Tan/MtL characterization

**FIGURE 8:**
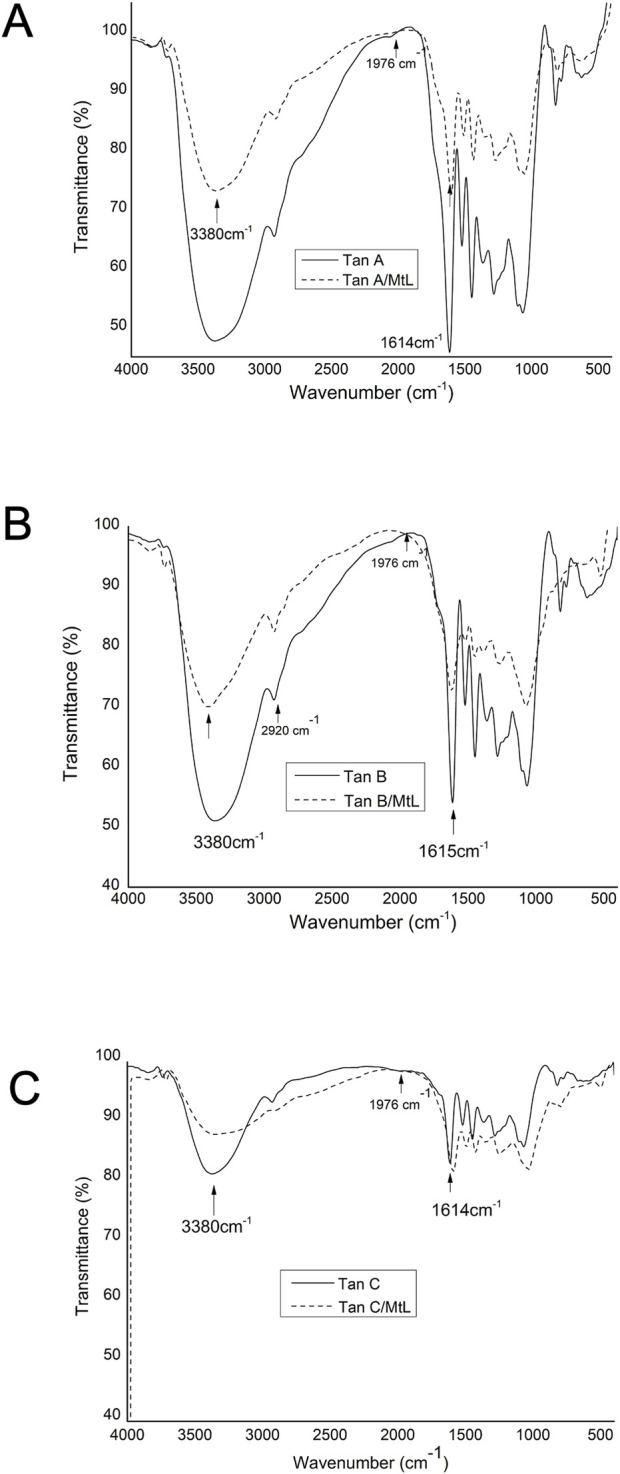
Comparison of FT-IR spectra of Tan A and Tan A/MtL **(A)**, Tan B and Tan B/MtL **(B)**, and Tan C and Tan C/ML **(C)**.

The reaction products of the enzymatic reaction of the tannins were characterized by FT-IR (Figure 8) under the same conditions as the raw tannins. This analysis was used to identify the types of bonds each tannin forms in response to the MtL enzyme. Figures 8A–C compare the FTIR spectra before and after the enzymatic reaction for Tan A, B, and C, respectively. The main difference observed in these spectra is the decrease of the band around 3,380 cm^−1^ corresponding to the stretching vibration of -OH groups. This decrease indicates the reduction of phenolic content due to the enzymatic reaction.

Additionally, for Tan C/MtL (Figure 8C), there is an increase in the band around 1614 cm^−1^ corresponding to the stretching vibration of the C=O bond, which can be attributed to oxidation processes carried out by the enzyme to produce quinones and semiquinones. This decrease in the intensity of the band associated with the -OH groups and the increase in the band associated with the C=O bonds is consistent with the reduction of phenol content observed during the enzymatic reaction.

**FIGURE 9:**
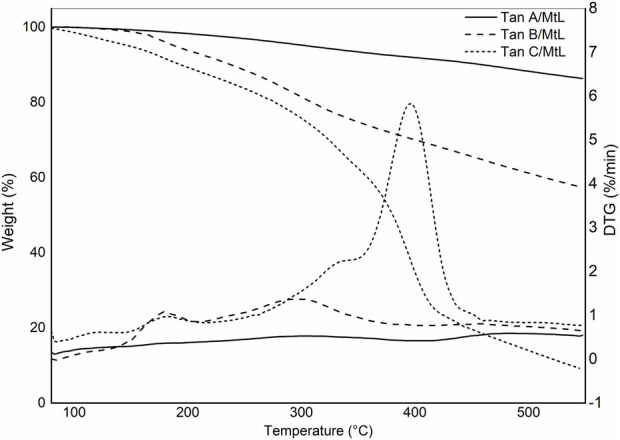
TGA and DTG profiles for tannins after enzymatic reaction.

Figure 9 illustrates the thermal decomposition profiles resulting from the enzymatic reaction of Tannins A, B, and C. Significant differences in the thermal stability of these polymers are evident. Tannin A/MtL demonstrated exceptional thermal stability, exhibiting negligible mass loss and achieving a residual mass of 86% at 550 °C. Tannin B only slightly increased its thermal stability, showing two distinct thermal events at 190°C and 300°C, resulting in a residual mass of 57%. Lastly, Tannin C/MtL exhibited the lowest thermal stability, characterized by a steep mass loss between 300°C and 400°C and a residual mass of 9%. The mass loss around 400°C has been attributed to the oxidation of phenolic compounds in the condensed phase of tannins (Xia et al., 2015). This would explain the increase in the intensity of this band for Tan C, confirming that in Tan C there were mainly oxidation processes of the phenolic species and not polymerization or cross-linking that would have improved the thermal stability of the final process.

Tan A, the apparent absence of carbohydrates and low-polarity compounds that could interfere with enzymatic polymerization seems to be a key factor contributing to the observed increase in viscosity and thermal stability. In contrast, for Tan C, these interfering compounds remain present, which likely promoted the oxidation of polyphenolic compounds in the tannins rather than enabling effective enzymatic polymerization. This could explain the lower thermal stability and viscosity observed in the polymer synthesized from Tan C, which is even lower than that of Tan B.

Furthermore, as regards the tannins before reaction with the laccase enzyme, which showed an exothermic absorption around 280°C (corresponding to catechin degradation due to the breakdown of the C2-C1′ bond of the C and B rings), for the tannins after enzymatic reaction, this band disappears and bands are observed at higher temperatures, indicating an increase in cross-linking and thermal stability. Considering the composition of the tannins, as shown in Figure 5, Tan A has a higher phenolic content and the lowest concentration of derived carbohydrates. This observation suggests that enzymatic polymerization would likely yield highly cross-linked polymers, explaining the observed improved thermal stability. When exposed to heat, this high thermal stability and carbon formation capacity make the polymer obtained from the enzymatic reaction of Tan A a material with great potential as a flame retardant.

In contrast, Tan B and Tan C display a lower phenolic abundance (for Tan B) and a significantly higher concentration of derived carbohydrates (for Tan C), implying the formation of less cross-linked polymers and polysaccharide residues. These factors contribute to lower thermal stability. Additionally, over this temperature pyrolytic degradation in this region involves fragmentation of inter-unit linkages, releasing monomeric phenols into the vapor phase, that may accelerate the degradation process (Tejado et al., 2007). Additionally, enzymatic depolymerization processes could be involved, which would similarly reduce the thermal stability of the material.

**FIGURE 10:**
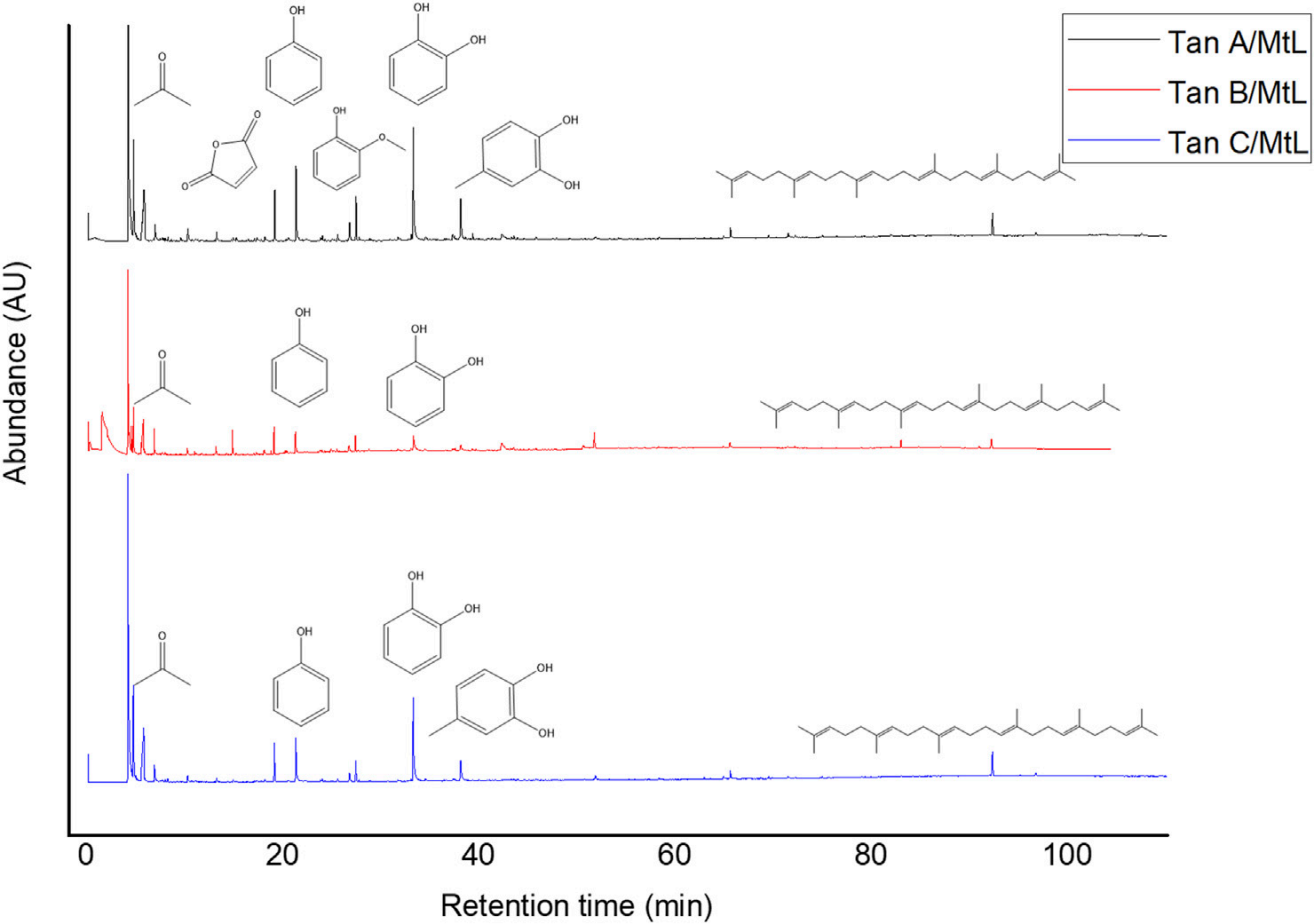
Py-GC/MS profiles for Tan A/MtL, Tan B/MtL, and Tan C/MtL.

Py-GC/MS analyses were also conducted under the same experimental conditions used for raw tannins at 550°C. For Tan A, the disappearance of the resorcinol signal post-reaction suggests that the enzymatic mechanism may target this compound (Figure 10). Similarly, the catechol and 4-methyl catechol decreased their relative content compared with raw Tan A but remained as the primary decomposition products detected in Tan A/MtL.

**FIGURE 11:**
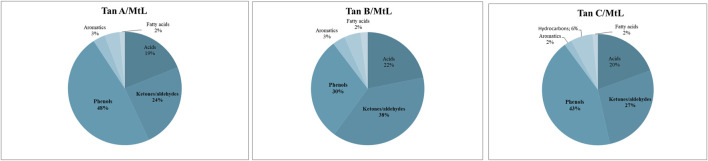
Primary decomposition product distribution for Tan A, B, and C after enzyme reaction.

Notably, organic compounds such as aldehydes, ketones, and carboxylic acids (43% of the relative amount) were detected in the Py-GC/MS analysis of Tan A/MtL but were in a minor content (9%) in raw Tan A. This indicates the possible occurrence of oxidation reactions (Figure 11). Tan B exhibited an increase in the relative content of carboxylic acids, ketones, and aldehydes, indicating significant oxidation of the sample, with relative content rising from 14% to 60%.

For Tan C/MtL, there was a notable increase in oxidized compounds such as carboxylic acids, ketones, and aldehydes, with their relative amount rising from 10% to 47%, indicating evident sample oxidation.

**FIGURE 12:**
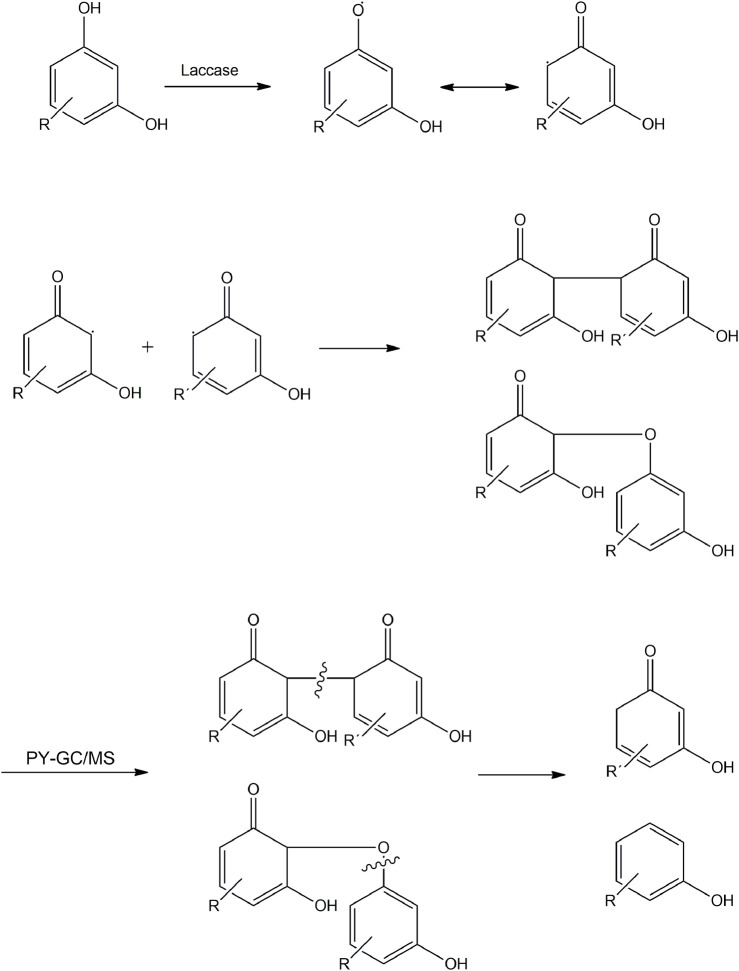
Proposed mechanism of enzymatic polymerization and thermal fractionation by Py-GC/MS.

Figure 11 shows that for all samples, a decrease in phenol content was observed after the enzymatic reaction, with the greatest reduction for Tan A (from 82% to 48% relative amount), followed by Tan C (from 57% to 43% relative amount), and finally for Tan B (from 38% to 30% relative amount) (Figure 11). This decrease in the phenol content, along with the decrease in the resorcinol content and the increase in C=O bonds for Tan A after the enzymatic reaction, suggests both oxidation processes and the formation of new ether bonds or substitutions in the aromatic ring to generate polymers, as evidenced by the increased viscosity observed. The proposed mechanism for these reactions starting from resorcinol is shown in Figure 12. It is proposed that the laccase enzyme-mediated polymerization of tannins occurred mainly through quinone radicals as intermediaries. The produced semiquinone radical could be transferred to another quinone ring, generating complex radical couplings that allow the formation of cross-linked polymers, which would explain the increase in the thermal stability of the polymer obtained for the Tan A (Yoshida et al., 2009).

For Tannin B, the changes associated with the enzymatic reaction could be attributed to oxidation or oligomerization reactions. For Tannin C, the changes can be attributed to oxidation processes, correlating with increased oxidized compounds such as aldehydes and ketones after the enzymatic reaction for these aqueous extracts. These results confirm the close relationship between the composition of each tannin-rich extract and its tendency toward enzymatic polymerization.

## 4 Conclusion

The study comprehensively investigates the structural impact of tannins on enzymatic polymerization, focusing on tannins extracted from *Pinus radiata* bark and polymerized using the MtL enzyme. The results indicated that a lower ash content enhances the tendency for enzymatic polymerization. Additionally, aqueous extracts with low molecular weight phenolic compounds did not exhibit a significant increase in viscosity, suggesting oligomer formation, as observed in Tan B. FT-IR and Py-GC/MS analyses of Tan A, which contained a higher initial phenol content, revealed the formation of new C-C and C-O-C bonds. During the enzymatic reaction, Tan A showed a reduction in phenolic content and an increase in viscosity, indicating a strong tendency toward enzymatic polymerization. The polymer derived from Tan A exhibited significantly improved thermal stability, highlighting its potential application as a temperature-resistant material make it the most suitable candidate for producing high-performance, eco-friendly polymeric materials with potential applications in flame retardancy. Further research could explore optimizing tannin extraction and purification processes to enhance polymerization efficiency and material properties.

## Data Availability

The original contributions presented in the study are included in the article/Supplementary Material, further inquiries can be directed to the corresponding authors.
