# Supplementary material for: Exploring tannin structures to enhance enzymatic polymerization
Source: Front Chem. 2025 Mar 4;13:1555202. doi: 10.3389/fchem.2025.1555202 (PMC11913842; doi:10.3389/fchem.2025.1555202)
Supplement: Supplementary file 1 [file DataSheet1.docx]

***Supplementary Material***

Exploring Tannin Structures to Enhance Enzymatic Polymerization

Romina Romero^a^,*, Tihare González^b^, Bruno F. Urbano^b^, Cristina Segura^c^, Alessandro Pellis^d^, and Myleidi Vera^b^*

**
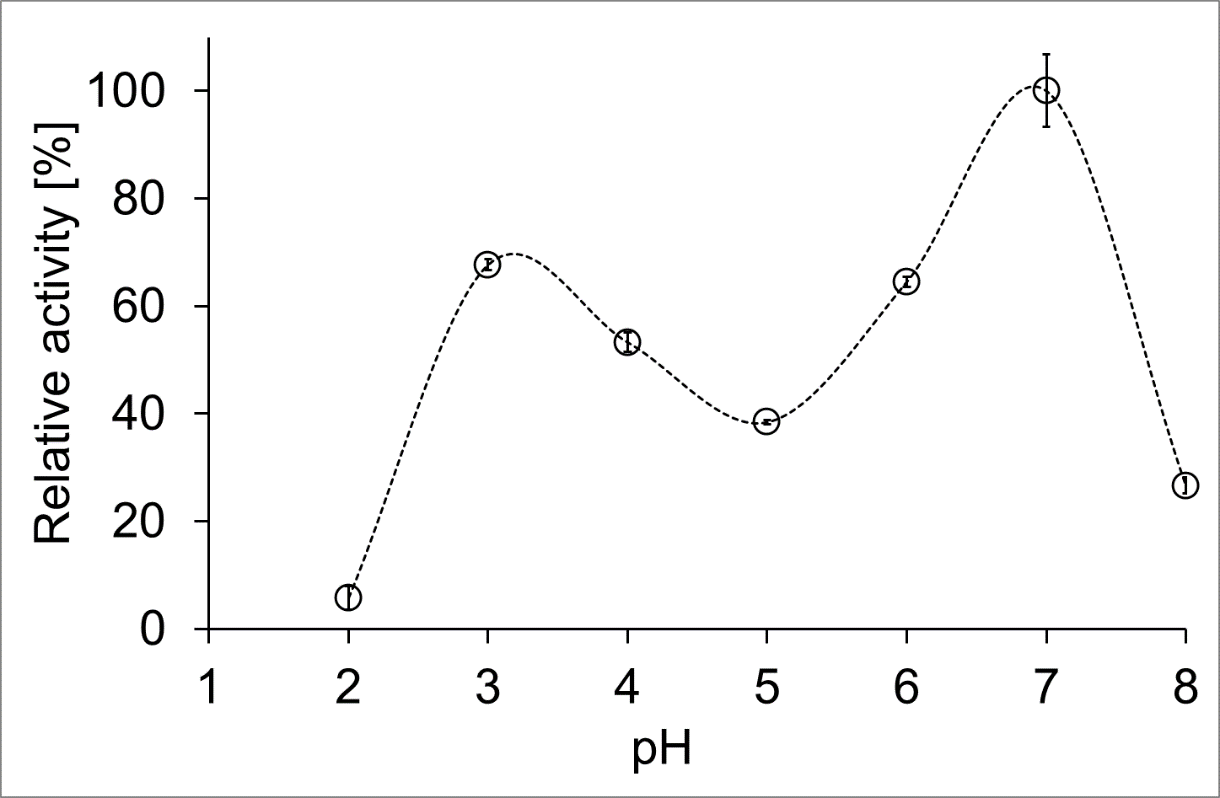
**

**Supplementary Figure 1.** Relative Myceliophthora thermophila laccase (MtL) enzyme activity at different pH values.


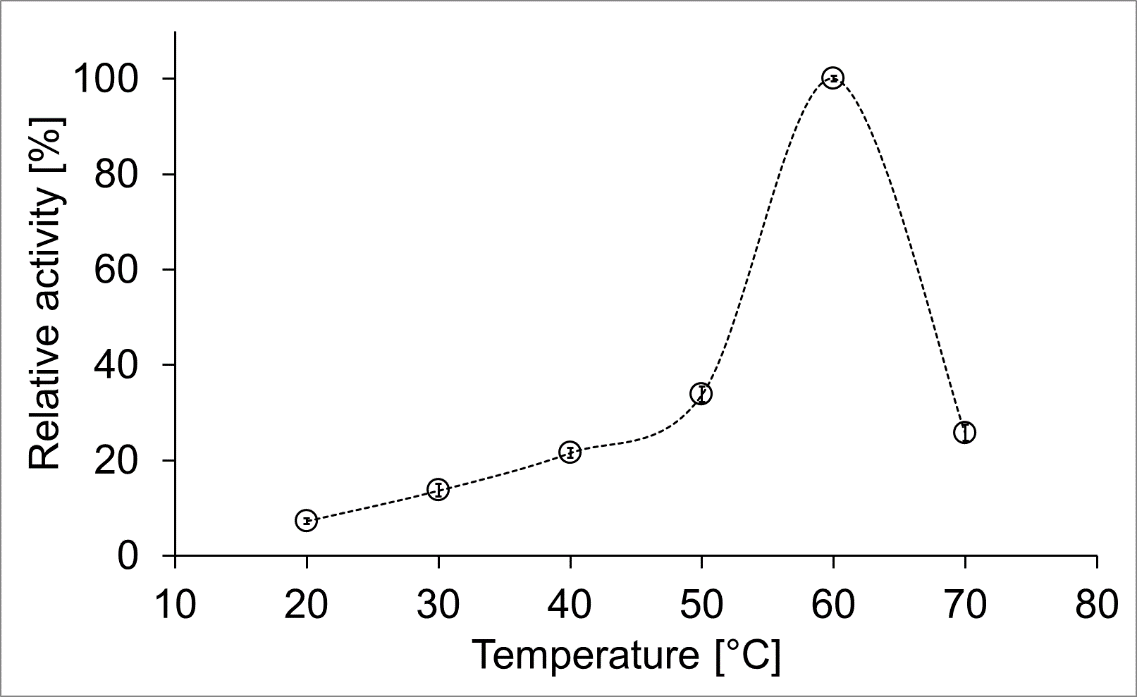


**Supplementary Figure 2.** Relative activity of Myceliophthora thermophila laccase (MtL) enzyme at different temperature values.

**Supplementary Table 1.** Comparative table of most important primary thermal decomposition compounds detected by Py-GC/MS at 550 °C for the studied tannins.

| **Compound** | **Classification** | **Tan A** | **Tan B** | **Tan C** |
| --- | --- | --- | --- | --- |
| Carbon dioxide | Light volatile | X | X | X |
| Acetone | Ketones/aldehydes | X |  | X |
| Isoprene | Hydrocarbon |  |  | X |
| Acetic acid | Acids | X | X | X |
| 2-Propanone,1-hydroxy- | Ketones/aldehydes | X | X |  |
| Toluene | Aromatic | X |  |  |
| Acetylacetone | Ketones/aldehydes | X |  |  |
| 5-Hydroxymethyl-2[5H] furanone | Furanes | X |  |  |
| Furfural | Furanes | X | X | X |
| Resorcinol | Phenols | X |  |  |
| 2,5-Furandione, | Furanes | X |  |  |
| Phenol | Phenols | X | X | X |
| o-Cymene | Aromatic | X |  |  |
| Phenol,3-methyl- | Phenols | X |  | X |
| 1,3-Benzenediol,2-methyl- | Phenols | X |  |  |
| Phenol,2-methoxy- | Phenols | X | X |  |
| 2-Methoxy-4-vinylphenol | Phenols | X |  |  |
| .alpha.-Methyl mannofuranoside | Carbohydrate-derived |  |  | X |
| Levoglucosenone | Carbohydrate-derived | X |  |  |
| Catechol | Phenols | X | X | X |
| D-Allose | Carbohydrate-derived |  |  | X |
| Phenol, 4-ethyl-2-methoxy- | Phenol | X |  |  |
| 2-O-Methyl-D-mannopyranosa | Carbohydrate-derived | X |  | X |
| 1,2-Benzenediol,4-methyl- | Phenol | X | X |  |
| 2-Methoxy-4-vinylphenol | Phenol | X |  |  |
| 4-O-Methylmannose | Carbohydrate-derived |  | X | X |
| 2-O-Methyl-D-mannopyranosa | Carbohydrate-derived |  |  | X |
| Pentadecanoic acid | Fatty acids | X | X | X |
| n-Hexadecanoic acid | Fatty acids | X | X | X |
| Octadecanoic acid | Fatty acids | X | X | X |
| Squalene | Hydrocarbon |  | X | X |
